# The Recent Applications of PLGA-Based Nanostructures for Ischemic Stroke

**DOI:** 10.3390/pharmaceutics15092322

**Published:** 2023-09-14

**Authors:** Jun Yan, Lei Huang, Juan Feng, Xue Yang

**Affiliations:** 1 Department of Neurology, Fushun Central Hospital, Fushun 113000, China; yanjun080212@163.com; 2 Department of Cardiac Function, Shengjing Hospital of China Medical University, Shenyang 110004, China; 3 Department of Neurology, Shengjing Hospital of China Medical University, Shenyang 110004, China

**Keywords:** ischemic stroke, drug delivery, theranostics, cell transplantation, imaging diagnosis

## Abstract

With the accelerated development of nanotechnology in recent years, nanomaterials have become increasingly prevalent in the medical field. The poly (lactic acid–glycolic acid) copolymer (PLGA) is one of the most commonly used biodegradable polymers. It is biocompatible and can be fabricated into various nanostructures, depending on requirements. Ischemic stroke is a common, disabling, and fatal illness that burdens society. There is a need for further improvement in the diagnosis and treatment of this disease. PLGA-based nanostructures can facilitate therapeutic compounds’ passage through the physicochemical barrier. They further provide both sustained and controlled release of therapeutic compounds when loaded with drugs for the treatment of ischemic stroke. The clinical significance and potential of PLGA-based nanostructures can also be seen in their applications in cell transplantation and imaging diagnostics of ischemic stroke. This paper summarizes the synthesis and properties of PLGA and reviews in detail the recent applications of PLGA-based nanostructures for drug delivery, disease therapy, cell transplantation, and the imaging diagnosis of ischemic stroke.

## 1. Introduction

With the advent of “global aging”, stroke is the world’s second-leading cause of death, as well as the third-greatest cause of disability [1]. Each year, 12.2 million cases of stroke occur worldwide, and 101 million people suffer from multiple stroke sequelae such as paralysis, aphasia, and cognitive impairment [2]. Ischemic stroke accounts for nearly 87 percent of all stroke cases [3]. Ischemic stroke is defined as a disruption in blood flow to the brain tissue, resulting in ischemia, hypoxia, necrosis of brain tissue, and clinically equivalent neurological impairments. The Trial of Org 10,172 in Acute Stroke Treatment (TOAST) classification is the most widely used etiological classification system for ischemic stroke [4]. The TOAST system consists of five major categories: large artery atherosclerosis, cardioembolism, small artery occlusion, stroke of other determined cause, and stroke of undetermined cause [5]. The treatment of ischemic stroke mainly includes intravenous thrombolysis, mechanical thrombus removal, and many neuroprotective agents. Due to the low BBB passing rate [6], short half-life [7], poor stability [8], low targeting [9] and large side effects of drugs [10], the therapeutic effect on ischemic stroke is limited. Therefore, there is a need to develop novel approaches for the treatment and diagnosis of ischemic stroke.

Nanomaterials have been widely used in ischemic stroke research and are regarded as having promising applications [11]. Poly (lactic acid–glycolic acid) copolymer (PLGA) is an outstanding example [12] and has been extensively explored for the treatment and diagnosis of ischemic stroke. PLGA, a Food and Drug Administration (FDA)-approved polymer, can be converted to carbon dioxide and water in the human body, which is biocompatible and almost non-toxic [13]. It can be fabricated into nanoparticles (NPs), nanocarriers, nanoscaffolds, and various other nanostructures as required.

**Figure 1:**
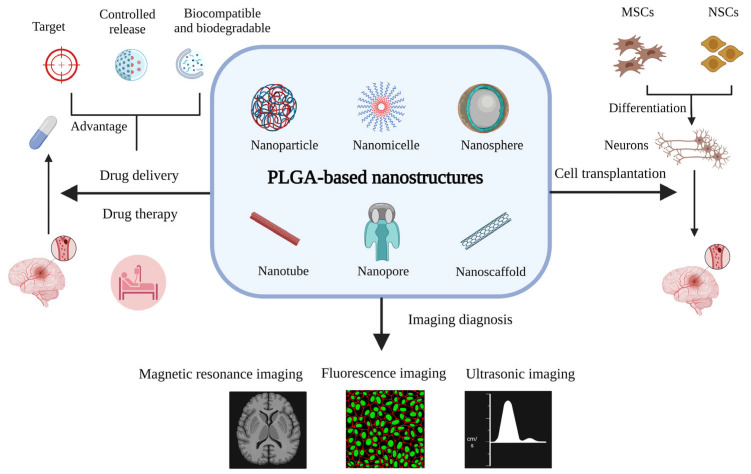
The applications of PLGA-based nanostructures for ischemic stroke (created with BioRender.com https://app.biorender.com/illustrations/6464d745074574a0c1d2f9fc accessed on 5 June 2023).

PLGA carriers have strong advantages in drug delivery. They can control the release of drugs, thereby prolonging their half-life and improving their bioavailability [14]. Drugs or bioactive ingredients can be targeted for absorption and prevent adverse reactions by crossing the BBB with PLGA carriers [15]. When used in conjunction with the targeted fragment, this method can precisely direct treatment to the site of a lesion in the brain [9]. PLGA scaffolds play a crucial role in cell transplantation [16], nerve regeneration [17], and tissue repair [18]. Ischemic stroke can be diagnosed and evaluated with greater precision when PLGA-based nanostructures are combined with staining markers or magnetic particles to allow for real-time tracking while imaging equipment observes [19]. In addition, the study of PLGA in gene therapy [20] and basic research [21] has also been reported. Nevertheless, there are few reviews on the application to PLGA nanostructures in ischemic stroke. This review will focus on this topic from an interdisciplinary perspective (Figure 1).

## 2. A Brief Introduction to PLGA and PLGA-Based Nanostructures

### 2.1. Synthesis and Properties of PLGA

**Figure 2:**
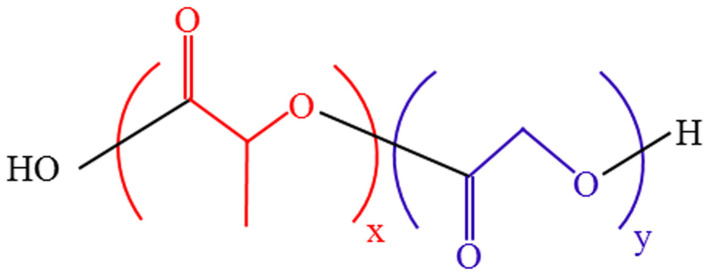
PLGA chemical structure.

PLGA is usually generated by polymerizing constituents of glycolic acid (GA) and lactic acid (LA) monomers [22] (Figure 2). Polycondensation and ring-opening polymerization are the primary synthesis methods for PLGA copolymer [23,24]. By adjusting the ratio of LA to GA (LA:GA) in the polymerization process, various PLGA copolymers can be produced [25]. Thus, various PLGA copolymers have different properties, such as strength, solubility, crystallinity, rate of hydration, rate of hydrolysis, glass transition temperature, and rate of biodegradation [26]. The glass transition temperature of an amorphous PLGA copolymer is 45–55 °C, and it has a rigid chain structure [27]. PLGA can be dissolved in different solvents, such as acetone, chlorinated solvents, tetrahydrofuran, and ethyl acetate [28]. PLGA is biodegraded by hydrolyzing the ester bonds of lactic acid and glycolic acid and then metabolized through the Krebs cycle to produce non-toxic water and carbon dioxide [29]. Therefore, PLGA has been widely used in medicine and biology, especially drug delivery [30], tissue engineering [31,32], and imaging diagnosis [33]. PLGA biodegradation is closely related to drug release because of its role as a drug carrier. PLGA biodegradation is influenced by three major variables [34]: First, the intrinsic physicochemical features of PLGA polymers and medicines [35,36]. Second, certain processing factors such as the polymer composite size and form [37], drug loading [38], and monomer ratio [39]. The 50:50 LA:GA ratio has the fastest degradation rate [28]. PLGA degradation also depends on the monomer sequence, and randomly arranged PLGA degrades faster than ordered PLGA [40]. Third, environmental parameters that influence release include temperature, pH, the in vitro/in vivo environment, etc., [25,28,41]. Multiple structural alterations can be applied to PLGA to optimize its functions as needed. For example, various PLGA copolymers are combined with polyethylene glycol (PEG), which can be combined with a hydrophilic group to enhance water solubility and prolong circulation time. In addition, they can be attached to particular ligands through covalent conjugation or electrostatic interaction to enhance their capacity for targeted drug delivery. The surface is functionalized by polyethylene glycol to confer PLGA NPs with “stealth” qualities, which are extremely significant. In other words, pegylation improves the pharmacokinetic behavior of PLGA NPs by shielding them from conditioning and phagocytosis [42,43].

### 2.2. PLGA-Based Nanostructures and Their Formulation

Various terms have been used to describe nanostructures, including NPs, nanowires, nanomicelles, nanoscaffolds, nanomembranes, nanotubes, nanocarriers, nanofibers, nanosystems, and so on. Due to their small size, high plasticity, high bioavailability, and biodegradability, PLGA-based nanostructures have been intensively studied in tumor [44], stroke [14], cardiovascular diseases [31], and autoimmune diseases [45]. Although there are many types of PLGA-based nanostructures, each has a specific formula, and the procedure is relatively simple. They can be synthesized by a variety of different routes, such as nanoprecipitation [46], salting out [47], emulsification–solvent evaporation [48], emulsification–solvent diffusion [49], spray drying [50], and electrospinning [51].

## 3. Applications of PLGA-Based Nanostructures in Drug Delivery and the Treatment of Ischemic Stroke

### 3.1. Mechanism of PLGA-Based Nanostructures Crossing the BBB

The BBB is a dynamic interface between the central nervous system and the blood circulation system. It controls how substances move between the blood and the brain and it keeps the body in balance [52]. The BBB is mainly composed of capillary endothelial cells, basement membrane, pericytes, astropodal cells, and tight junctions (TJs). To meet the energy supply and nutritional requirements of brain tissue, the BBB allows the selective passage of water, some gases, electrolytes, and major nutrients. As a natural barrier, the BBB protects the brain from damage [53], but it also acts as a barrier to therapeutic drug passage [54]. However, many drugs are unable to cross the BBB, and they cannot reach the lesion effectively.

**Figure 3:**
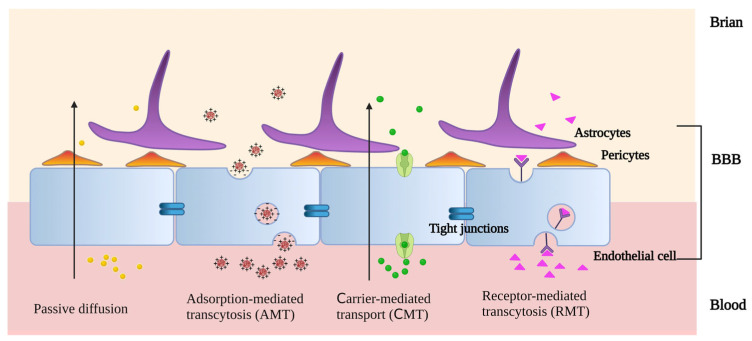
The methods by which PLGA-based nanostructures penetrate the brain (created with BioRender.com. https://app.biorender.com/illustrations/646086fd04c4a384e3d4472e accessed on 5 June 2023).

As drug carriers, PLGA-based nanostructures can penetrate the brain in a variety of ways (Figure 3). On the one hand, PLGA-based nanocarriers can go through the BBB passively, and most of them are unmodified. Passive diffusion, depending on size, is the primary mechanism, but brain absorption is low [15]. The ability of PLGA NPs with a different LA:GA to function as nanocarriers for drug transfer varies as well. Comparing the neuronal uptake of curcumin, NPs-Cur 50:50, and NPs-Cur 65:35 revealed that SK-N-SH cells had a greater uptake of NPs-Cur 50:50 than NPs-Cur 65:35 or free curcumin [55]. 

On the other hand, PLGA-based nanocarriers can pass through the BBB via active targeting, which is a selective and specific mode of transit. This frequently occurs with modified PLGA-based nanocarriers through adsorption-mediated transcytosis (AMT), carrier-mediated transport (CMT), and receptor-mediated transcytosis (RMT). In AMT systems, cationic modification is a typical technique. Positive charges are added to the surfaces of PLGA-based nanocarriers, which interact electrostatically with the negatively charged areas of the endothelium membranes to increase brain uptake. In the acidic pH of tumors, PAMAM dendritic macromolecules can protonate free amino groups. Vimalkumar et al. used the adsorptive cytophagy of dendritic cationized albumin encapsulated in PLGA NPs (dCatAlb-pdnp) to bypass the BBB and achieve the efficient delivery of doxorubicin (DOX) to glioblastoma [56]. There are a variety of transporters in the BBB. Their substrates are mostly membrane-permeable molecules, such as glucose, amino acids, and membranophilic peptides. With CMT, PLGA-based nanocarriers can efficiently cross the BBB and deliver medications to the brain through the modification of membrane-permeable compounds [57]. As a biocompatible surfactant, Pluronic F-68 was coated on the surface of PLGA NPs to considerably increase their penetration. This allowed for a greater amount of medication loaded by NPs to penetrate the central nervous system. When darunavir (DRV), an antiviral medication, was encapsulated in PLGA NPs, its penetration of the blood–brain barrier increased from 9 ± 0.1% to 38.0 ± 2.1% [58]. RMT is based on the specific binding of the ligand to the receptor [59]. GPIIb-IIIa is a glycoprotein receptor on activated platelet membranes that is generated at the onset of the pathological thrombotic process. It has been demonstrated that peptides with a cyclic arginine–glycine–aspartic acid structure (cyclic Arg-Gly-Asp, cRGD) have increased binding affinity for GPIIb-IIIa. cRGD-modified PLGA NPs can facilitate the binding of thrombolytic medications to activated platelets via RMT, thereby enhancing thrombolysis rates [60]. Brain endothelial cells express a wide variety of receptors to ensure that only the desired ligand is taken up. DAS peptides are a type of ligand that was designed to be specific to the alpha-7 nicotinic receptors, which are found on the surface of brain endothelial cells. Recent research has shown that DAS-conjugated PLGA NPs are an effective carrier for the transport and RMT-mediated release of large hydrophilic molecules to the BBB [61]. PEG-PLGA NPs were modified with an acetylcholine receptor-binding specific ligand (RVG29 peptide) in order to improve the targeting of chemotherapeutic agents. An enzyme immunoassay analysis revealed that the number of receptors on the surface of glioma cells was 2.04-fold higher than that of non-malignant cells [59]. PLGA-based nanocarriers can be endocytosed and cross the BBB easily with appropriate ligand attachments. Drug delivery via BBB is facilitated by PLGA nanocarriers, enabling more precise treatment. 

### 3.2. Applications of PLGA-Based Nanocarriers in Ischemic Stroke Treatment

Several mechanisms are available for treating ischemic stroke, including thrombolytic therapy, anti-oxidative stress and anti-apoptosis, anti-inflammation, the inhibition of neuroexcitatory toxicity, and supplementation with neurotrophic factors [62]. In recent years, PLGA nanocarriers have shown a remarkable potential for ischemic stroke treatment. The role of PLGA nanocarriers in drug delivery and ischemic stroke treatment is discussed below based on different mechanisms of treatment, as shown in Table 1.

#### 3.2.1. Thrombolytic Therapy

Acute ischemic stroke treatment includes intravenous thrombolysis and interventional therapy [83]. Interventional therapy is an invasive treatment that is expensive, risky, and unacceptable for most people [65,84]. Intravenous thrombolytic therapy is non-invasive; thus, optimizing intravenous thrombolytic therapy is of paramount significance in clinical practice [85,86]. The US FDA approved t-PA as a thrombolytic drug for acute ischemic stroke as early as 1996. It is currently the only drug approved by the FDA at present [87]. The use and treatment of rtPA have been limited because of the following factors: a narrow therapeutic time window (usually within 4.5 h), a short half-life, a weak affinity for the thrombus, and bleeding complications [88]. In recent years, in vivo studies have demonstrated that PLGA-based NPs are effective as thrombolytic agent carriers. Masumeh et al. used tPA to produce tPA-PEG-PLGA NPs. This enabled tPA to stay in circulation longer in vitro and improved its ability to target itself and break up clots. It was found that the thrombolytic activity of PLGA NPs was 1.5–4 times that of free tPA solution, and the thrombolytic activity of PEG-PLGA NPs was 2–6 times that of PEG-PLGA NPs. Moreover, the engineered NPs had an excellent biocompatibility and biodegradability, and their formulation method was easy, cheap, and safe [63]. Chen et al. designed peptide/rtPA conjugated PLGA magnetic NPs (pPMNP-rtPA) by co-immobilizing rtPA and a fibrin-avid peptide to PLGA magnetic NPs (PMNP). Compared with free rtPA, pPMNP-rtPA could shorten clot lysis time by 40% at the same drug dose. This might significantly reduce the thrombolysis bleeding risk. Iron oxide magnetic NPs in PMNP enable drug delivery. When conjugated to pPMNP-rtPA, rtPA has the same clot-lysis efficiency. Meanwhile, the fibrin-avid peptide enhances rtPA’s affinity for the thrombus. The dual target pPMNP-rtPA is preferable to free rtPA during in vitro and in vivo thrombolysis for its magnetic guiding and fibrin-binding characteristics [64]. Liu et al. prepared a phase-change nanoparticle targeting thrombus fibrin. The shell of the nanoparticle is attached to CREKA peptides, which may facilitate the targeted aggregation of NPs on the thrombus. The core of the NP consists of rtPA and perfluorohexane (PFH). As a phase-change material, PFH is transformed from a liquid to a gaseous state under low-intensity focused ultrasound (LIFU). At the thrombus site, this process can act as an excavator and release rtPA specifically. Compared to either method alone, this combined thrombolytic approach (mechanical thrombolytic and medication thrombolytic) is superior because it is more effective, safer, and can be monitored in vivo [65]. Urokinase is also a regularly utilized intravenous thrombolytic medication in clinical practice. Intravenous thrombolysis with urokinase within 6 h after the onset of acute ischemic stroke is considered beneficial. It is less expensive than rtPA [49] but has a higher risk of extracranial bleeding [89]. Indocyanine green (ICG) is a photosensitizer used in clinical fluorescence imaging and is a crucial element in phototherapy. In a recent study, the ICG complex of uPA (IGC@uPA) was developed, and a cRGD-modified PEG-PLGA NP was used as its carrier to form an enzyme–phototherapy synergistic thrombolytic platform. The nanocarriers produced can boost the thrombolysis rate and lower the risk of bleeding considerably. According to the results of the in vitro thrombolysis experiments, the thrombolysis rate was 72% in the cRGD-ICG-uPA NPs group and 60% in the free uPA group. The average time for hemostasis in the cRGD-ICG-uPA NPs group was around 200 s, while in the free uPA group it was around 600 s [66].

#### 3.2.2. Anti-Oxidative Stress and Anti-Apoptosis

**Figure 4:**
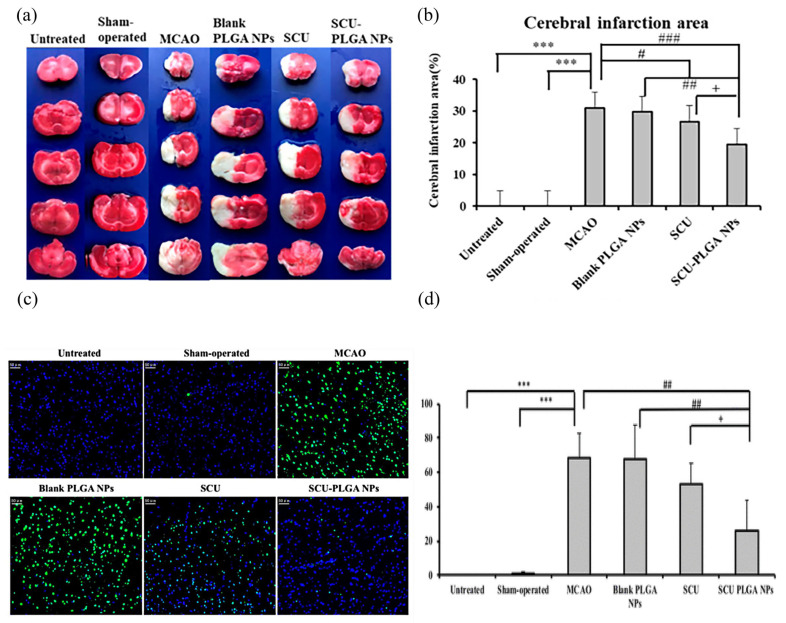
SCU-PLGA NPs delivered intravenously alleviated cerebral ischemia in rats and decreased neuronal apoptosis after cerebral ischemia. Brain slices stained with TTC are shown in (**a**) representative images; the cerebral infarction region is analyzed in (**b**); and (**c**) representative images of TUNEL-positive cells are shown. The apoptotic cells were labelled with TUNEL (green), and the nucleus was stained with DAPI (blue). Scale bar: 50 μm. (**d**) Apoptotic cell percentage in the brains of MCAO rats (n = 3). Compared with the untreated or shamoperated groups, *** *p* < 0.001; compared with the MCAO group or blank PLGA NPs, # *p* < 0.05, ## *p* < 0.01, and ### *p* < 0.001; and compared with SCU, ^+^ *p* < 0.05. Data shown as mean ± SD. Adapted with permission from [68]. Copyright (2022) American Chemical Society.

The benefits of thrombolysis in treating acute ischemic stroke to achieve vascular revascularization are self-evident; however, cerebral ischemia-reperfusion (CIR) can induce the production of harmful reactive oxygen species (ROS), which in turn trigger oxidative stress (OS), leading to the breakdown of the blood–brain barrier and the accelerated apoptosis of nerve cells [90,91,92]. As an antioxidant and regulator of brain cell apoptosis, edaravone is broadly applied in the clinical treatment of acute ischemic stroke [93]. Edaravone micelles are generated by combining mPEG-bPLGA copolymers with edaravone. Sharifyrad et al. used the human neuroblastoma SH-SY5Y cell line to create an in vitro ischemia model. In this model, edaravone micelles were more effective than free edaravone at lowering the lactate dehydrogenase (LDH), nitric oxide (NO) activity, intracellular reactive oxygen species (ROS), cell apoptosis rate, and pro-apoptotic gene (Bax) expression and raising anti-apoptotic gene (HSP70 and Bcl-2) expression [67]. Several traditional Chinese remedies or their components possess anti-oxidation and anti-apoptosis properties, which are advantageous in the treatment of ischemic cerebrovascular disorders. Scutellarin (SCU), a flavonoid extracted from Erigeron breviscapus, has pharmacological properties including anti-oxidation and anti-apoptosis, as well as considerable results in cardiovascular and cerebrovascular illnesses [94,95,96]. In one study, SCU was encapsulated in PLGA NPs to form SCU-PLGA NPs. In a rat model of CIR, the diseased rats treated with saline or blank PLGA NPs exhibited comparable neurological scores of 2.50 ± 0.55 and 2.33 ± 0.52, respectively. On the contrary, both SCU groups showed a better performance since lower neurological scores were recorded in the diseased rats treated with SCU-PLGA NPs (1.33 ± 0.52) and free SCU (1.83 ± 0.41); the former was lower than the latter. The infarct size of the sick rats treated with SCU-PLGA NPs (19%) was smaller than that of the rats treated with free SCU (27%) [68]. It was also observed that SCU-PLGA NPs greatly extended the release curve and blood circulation duration of SCU, as well as preventing nerve cell apoptosis, as shown in Figure 4. TanIIA (tanshinone IIA) possesses multiple pharmacological properties, including antioxidant, anti-inflammatory, and autoimmune regulation [97,98,99]. However, it is poorly soluble and has a short half-life. PLGA NPs as the drug carrier for Tan IIA could evidently increase its bioavailability and therapeutic efficacy. The intracisternal injection of drug-loaded Tan IIA NPs (Tan IIA-NPs) reduced the cerebral infarct volume and motor deficits in a porcine ischemic stroke model. The T2W sequences 24 h after stroke also showed a reduction in the acute ischemic lesion volume (9.54 ± 5.06 vs. 12.01 ± 0.17 cm^3^) in Tan II-NP-treated pigs compared with the Tan IIA-treated pigs. When Tan IIA-NPs were added to neural stem cells (NSCs) in the lab, the superoxide dismutase (SOD) activity decreased. TNF- αand IFN-γ were not detected in the Tan IIA-NPs-treated group compared with the positive control group, which had significantly elevated levels of TNF- α and IFN-γ (128.03 ± 26.21 and 45.92 ± 1.02 pg/mL, respectively). These results suggest that Tan IIA-NPs may treat stroke through an antioxidant mechanism, and the results are significantly superior to free Tan IIA treatment [69]. The design of NL-1 was inspired by the thiazolidinedione structure of pioglitazone. Pioglitazone is an anti-diabetic drug with neuroprotective effects. As a peroxisome proliferator-activated receptor γ (PPARγ) agonist, pioglitazone decreases oxidative stress and ROS formation [100]. In an ischemia-reperfusion rat model, NL-1 treatment dramatically enhanced survival and improved functional outcomes. Moreover, it lowered oxidative stress and apoptosis in brain cells while improving blood–brain barrier function [101]. When NL-1 was loaded with PLGA NPs, this therapeutic efficacy was again obtained at a dosage 40 times lower than that of free NL-1 [70]. Exendin-4 (Ex-4) is a glucose-lowering drug that also has therapeutic effects on cerebrovascular diseases [102]. Nevertheless, its therapeutic effectiveness is constrained by its short half-life and poor biological activity. Ex-4 was packed onto PLGA NPs to generate Ex-4-loaded poly(d,l-lactide-co-glycolide) NPs (PEx-4). PEx-4 significantly reduced brain edema, ROS activity, and neuronal apoptosis in IR diabetic rats compared to Ex-4 treatment. Moreover, PEx-4 had a longer-lasting hypoglycemic impact than Ex-4. The study demonstrates that PEx-4 has a potent antioxidant activity and sustained bioavailability, suggesting that it may ameliorate IR-induced brain damage in diabetic rats by inhibiting OS and nerve cell apoptosis [71]. Mitochondrial malfunction and ROS overproduction damage neurons in CIR [103]. Curcumin can prevent the oxidative damage due to CIR, but free curcumin has a low BBB passage rate and a poor bioavailability. To address the aforementioned issues, PEG-PLGA NPs (NC) containing curcumin were developed. In the CIR model, the ROC values of rats in the no-pretreatment group, the free curcumin pretreatment group, and the NC pretreatment group were 293 ± 23, 251 ± 27, and 121 ± 13, respectively. NC preconditioning can be made more effective by decreasing ROS production, thereby decreasing ROS-induced mitochondrial dysfunction and neuronal apoptosis [72].

#### 3.2.3. Anti-Inflammation

Inflammation is a critical factor in arteriosclerosis and thrombosis and plays a significant role in stroke onset and progression [104]. The use of NPs as carriers to deliver anti-inflammatory medications into the central nervous system has become a novel direction in the treatment of ischemic stroke.

In one study, RVG29 peptide-modified polyethylene glycol–polylactic acid –glycolic acid copolymer NPs (PEG-PLGA RNPs) were used to carry baicalin (BA), which was chosen as a neuroprotective agent. Compared with the BA buffer group, the BA-PEG-PLGA NP group, and the BA-PEG-PLGA RNPs with intravenous administration group, the BA-PEG-PLGA RNPs group had the smallest infarct volume (19.28%, 17.28%, 15.00%, and 11.19%, respectively) after treatment. The neurological deficit score due to ischemic brain injury was the smallest (0.87) in the animals in the BA-PEG-PLGA RNPs group, compared to the neurological deficit score of the BA buffer group, the BA-PEG-PLGA NP group, and the BA-PEG-PLGA RNPs with intravenous administration group (2.33, 1.80, and 1.33, respectively). At the same time, following treatment with BA NPs, the serum levels of IL-1β, IL-6, and TNF-α in rats with cerebral ischemia decreased. It is suggested that nanocarriers facilitate the smooth and continuous release of BA, which improves its bioavailability. Effective drug delivery permits the anti-inflammatory benefits of the neuroprotective agent to be completely unleashed [73]. Liu et al. utilized PLGA NPs modified with α-lipoic acid as drug carriers to load the neuroprotective cannabidiol. Due to the chemotaxis of neutrophils to the infarct core and their affinity for inflammatory cytokines, PLGA NPs might be wrapped around neutrophil membranes to target the infarct core. At the same time, cannabinol could also neutralize harmful chemicals in the core area of cerebral infarction. As a result, the treatments of CBD, NM-NP/CBD, and LA-NM-NP/CBD all reduced the infarct volumes of middle cerebral artery occlusion (MCAO) rats to 74.2%, 38.8%, and 26.3%, respectively, 24 h after injection, among which LA-NM-NP/CBD exerted the most significant effect on diminishing the infarct area. Furthermore, the neurological deficit score of MCAO rats treated with saline was evaluated at 3.4, suggesting a severe neurological deficit. Nevertheless, MCAO rats treated with CBD, NM-NP/CBD, and LA-NM-NP/CBD all improved the neuro-functional deficiency to different extents. Specifically, the neurological score of MCAO rats in the LA-NM-NP/CBD treated group decreased to 1.2, showing the most significant improvement in neurological recovery among all groups [74].

Microglia cells are essential cells that promote inflammation following an ischemic stroke. When ischemic stroke occurs, microglia are activated and polarized into either a proinflammatory M1 phenotype or an anti-inflammatory M2 phenotype [105]. Proinflammatory M1 microglia are a prominent source of inflammatory cytokines that induce endothelial cell necrosis and blood–brain barrier leakage following ischemic stroke [106]. Elevated neuroinflammation and cerebral edema contribute to a dismal prognosis [107]. Controlling the polarization direction of microglia cells to lessen inflammatory damage is expected to be an innovative therapeutic strategy for ischemic stroke. In a model of ischemic stroke, perampanel-loaded PLGA NPs boosted microglial cell polarization towards the M2 phenotype and inhibited the release of pro-inflammatory cytokines, such as TNF-α, IL-1β, and IL-6 [75]. Kim et al. demonstrated that PLGA NPs loaded with PINK1 siRNA (PINK1 NPs) could be selectively taken up by microglia cells, increasing the expression of an anti-inflammatory state in microglia cells [76].

#### 3.2.4. Inhibition of Neuroexcitatory Toxicity

It is well known that glutamate-driven excitotoxicity is one of the classical mechanisms of injury following an ischemic stroke. During an ischemic stroke, ion transporter malfunction and the alteration of ion balance result in decreased glutamate release and reuptake and the overactivation of the N-methyl-D-aspartate receptor (NMDAR), which accelerates neuron death [108]. Estradiol prevents glutamate-induced neuron necrosis by regulating small albumin and intracellular calcium ions [109]. Estradiol-containing PLGA NPs were more neuroprotective against glutamate-induced excitotoxic neuronal death than estradiol alone [77]. When an ischemic stroke occurs, matrix metalloproteinase-9 (MMP-9) expression is increased, which is intimately associated with excitotoxicity, neuronal injury, and blood–brain barrier breakdown [110]. Many studies focus on decreasing MMP-9 as a target for ischemic stroke [111,112]. Matrix metalloproteinase tissue inhibitor -1 (TIMP-1) is an endogenous inhibitor of MMP-9. Chaturved et al. used the recombinant mouse TIMP-1 to counteract excitatory toxicity in a hippocampal tissue sheet culture (OHC) model induced by sea human acid (KA). In order to provide a gradual release effect for TIMP-1, it was coupled with PLGA NPs to form TIMP-1 PLGA NPs. In this way, the neuroprotective impact of inhibiting MMP-9 activity would be more pronounced [78]. Erythropoietin (EPO) rescues neurons from glutamate excitotoxicity by regulating apoptosis and safeguarding astrocyte function [113,114]. PLGA NPs loaded with recombinant human erythropoietin (rhEPO) stabilized by sodium cholate (rhEPO-CH-NP) were less hazardous and had a greater capacity to protect neurons from glutamate excitotoxicity than rhEPO [79]. In a recent study, cholic acid was wrapped around EPO-loaded PLGA NPs (EPO-CA-nps) to help EPO to cross the BBB. When applied to a rat stroke model, the infarct volume generated by MCAO/R surgery was significantly decreased in the EPO and EPO-CA-NP groups compared with the Veh group (40.28 ± 6.13 and 33.79 ± 5.83 vs. 63.57 ± 3.75%), which were shown in the representative images of TTC-stained brain slices and the quantitative graph revealing the infarct volumes. During Garcia’s neurological scoring and the rotarod test, the EPO-CA-NP group maintained the highest score compared with the Veh group at postoperative days (POD) 1, 3, 5, and 7 (14.67 ± 1.53, 14.00 ± 1.00, 15.33 ± 1.53, and 15.00 ± 1.00 vs. 5.67 ± 0.58, 6.67 ± 0.58, 6.00 ± 1.00, and 10.67 ± 0.58, respectively). Notably, Garcia’s neurological test revealed that the sensorimotor performances of the EPO-CA-NP group were significantly higher than those of the EPO group at all time points (vs. 11.33 ± 0.58, 9.67 ± 1.53, 10.33 ± 0.58, and 11.67 ± 0.58 at POD 1, 3, 5, and 7, respectively). Interestingly, the latencies to fall of the EPO-CA-NP group were significantly longer than those of the EPO group at POD 3, 5, and 7 (90.20 ± 22.21, 122.60 ± 51.74, and 300 ± 0.00 vs. 10.00 ± 2.24, 58.00 ± 43.17, and 107.60 ± 40.77 s at POD 3, 5, and 7, respectively). EPO-CA-NPs therapy dramatically improved motor and sensory function while decreasing cerebral infarction volume compared to EPO alone [115]. Indole-3-methanol (I3C) has been shown to have neuroprotective effects in animal models of ischemic stroke [116]. Unfortunately, I3C cannot cross the BBB, significantly reducing its therapeutic efficacy. The I3C-loaded PLGA NPs, stabilized with Tween 80 (T80), are precisely designed to compensate for this deficiency. When PC12 neurons were treated with either I3C or I3C-PLGA-T80-NPs, the latter resulted in a much better survival rate [80].

#### 3.2.5. Supplementing with Neurotrophic Factor

Neurotrophic factors are critical for neuronal survival as they promote neuronal proliferation, survival, and differentiation [117]. Neurotrophic factors regulate a variety of neurological activities in ischemic stroke, including neuroprotection [118], neuroplasticity [119], and vascular remodeling [120]. Several studies have shown that neurotrophic factors can be a treatment option for ischemic stroke [121,122,123]. Neurotrophic factors can be degraded by enzymes or formed into protein–antibody complexes, resulting in a decreased bioavailability, poor tissue distribution, and short half-life [124,125]. Nanomaterials have been used as drug-delivery systems to overcome these pharmacokinetic problems. Brain-derived neurotrophic factor (BDNF) is the predominant growth factor in the central nervous system. Kamarudin et al. developed NP-BDNF by combining BDNF and PLGA NPs. In a rat model of permanent middle cerebral artery occlusion (pMCAO), the total infarct volume was observed and measured in the stroke (204.95 mm^3^), BDNF-treated (210.10 mm^3^), and NP-BDNF-treated (107.58 mm^3^) groups. The NP-BDNF-treated group showed a significant 1.9-fold decrease in infarct volume after the treatment with NP-BDNFs compared to the stroke group. The modified neurologic severity score (mNSS) remained significantly higher in the post-BDNF-treated group by 14-fold (*p* < 0.001), and it was improved in the post-NP-BDNF-treated group by a 7-fold decrease (*p* < 0.001), when compared with sham-operated rats. Therefore, therapy with NP-BDNF decreased the infarct size, the severity of limb paralysis, and the expression of brain-tissue damage markers compared to BDNF administration [81]. Jaclyn et al. created a BDNF delivery system using a hydrogel containing hyaluronic acid, methylcellulose (HAMC), and PLGA NPs and applied it directly to the epidermis directly above the stroke. The electrostatic interaction between the positively charged BDNF and negatively charged PLGA NPs enabled slow and sustained drug release without encapsulation. After drug delivery, BDNF was detectable in the tissue for 21 days. Sodium hyaluronate (HA) protects nerves due to its anti-inflammatory properties. As a result of the delivery of BDNF using the PLGA-HAMC vector to the lesion brain tissue, the total stroke lesion volume (cortical and striatal combined) in the BDNF and vehicle groups was significantly less than that of the injury group (10.86 mm^3^, 12.22 mm^3^, and 24.38 mm^3^, respectively). The cortical lesion volume in the BDNF and vehicle groups was significantly decreased compared to the injury group (15.68 mm^3^, 17.09 mm^3^, and 31.48 mm^3^, respectively), while the striatal lesion volumes were similar across all groups (5.37 mm^3^, 5.43 mm^3^, and 7.32 mm^3^, respectively). Significant increases in synaptophysin expression were observed in the BDNF-treated animals compared with the vehicle groups and the injury group in the contralesional hemisphere regions of interest (ROI) R1(7.08 × 10^5^ µm^2^, 5.20 × 10^5^ µm^2^, and 4.77 × 10^5^ µm^2^, respectively) and R2 (7.18 × 10^5^ µm^2^, 5.13 × 10^5^ µm^2^, and 4.84 × 10^5^ µm^2^, respectively). This suggests that PLGA-HAMC carrying BDNF reduces the cerebral infarction volume and enhances synaptic plasticity contralateral to the lesion in the rat ischemic stroke model [82]

## 4. Applications of PLGA-Based Nanostructures in Cell Transplantation for Ischemic Stroke

Cell transplantation is a research hotspot in the field of ischemic stroke treatment. Stem cells have the advantage of low immunogenicity, can differentiate into many types of cells as needed, and can replace damaged brain cells, thus making them the first choice for cell transplantation [126]. There are various types of stem cells used in stroke research, such as neural stem cells and mesenchymal stem cells (MSCs).

Nerve stem cells (NSCs) can differentiate into neurons, oligodendrocytes, and astrocytes. Through cell replacement, NSCs can heal damaged brain tissue after a stroke and minimize the infarct size [126]. NSC are also able to control the hypoxic microenvironment in the brain through modulating inflammation and nourishing nerves [127]. The transplantation of NSCs has emerged as a promising strategy for treating ischemic stroke [128]. To repair stroke lesions, NSCs are employed in conjunction with suitable nanomaterials to maintain these cells in the infarct cavity and speed the development of new tissue [11]. Combining NSCs with appropriate nanomaterials could preserve these cells in the infarct lumen and accelerate new tissue formation. Composite neural materials are receiving more attention as a potential treatment for nerve injuries. NSCs were cultured on a PLGA/graphene oxide (GO) composite membrane, which was then subjected to electrical stimulation (ES). The data demonstrated that the PLGA/GO membrane possessed a desirable hydrophilicity, mechanical strength, and protein adsorption capacity. After ES was added to the PLGA/GO membrane for 3 d and 7 d, the results of the proliferation of NSCs for the PLGA/GO and PLGA + ES groups were significantly higher than those for the PLGA group (0.74 ± 0.07, 0.85 ± 0.03, and 0.61 ± 0.04, 3 d, respectively; 1.26 ± 0.02, 1.29 ± 0.04, and 1.11 ± 0.05, 7 d, respectively). In addition, the OD values (1.53 ± 0.04) for the PLGA/GO + ES group at all culture time points were the highest among the four groups at 7 d (*p* < 0.05), indicating that ES can be combined with the PLGA/GO conductive composite material to further enhance NSC proliferation on the material surface. The average neurite length result (62.4 ± 15.4 μm) in the PLGA group was significantly shorter than that in the other three groups (*p* < 0.05). The neurite lengths in the PLGA/GO group (97.5 ± 18.9 lm), the PLGA + ES group (137 ± 13.2 lm), and the PLGA/GO + ES group (179.8 ± 24.5 lm) showed an increasing trend, and the pairwise comparison results showed significant differences. The PCR results indicated that both the PLGA/GO and PLGA+ES groups significantly promoted NSC differentiation into neurons and, to some extent, inhibited NSC differentiation into astrocytes, although the two groups were not significantly different [129]. Modifying biomaterial surfaces is essential for increasing their bioactivity and stimulating repair processes for nerve regeneration. The growth factor insulin-like growth factor 1 (IGF-1) has neuroprotective and neurogenic properties. Qi et al. discovered that IGF-1 immobilized on PLGA/graphene oxide (GO) nanofibers greatly increased NSC survival, proliferation, and differentiation. This research studied NSC proliferation on Day 7 in PLGA, PLGA/GO, and PLGA/GO/IGF-1 (10, 100, 500 ng mL^−1^). The OD value of the PLGA/GO group was much higher than for PLGA. The three PLGA/GO/IGF-1 groups showed a much higher optical density (OD) than PLGA and PLGA/GO. PLGA/GO/IGF (at all three doses) outperformed PLGA/GO in an H_2_O_2_-stimulated microenvironment. IGF-1-modified PLGA/GO nanofibers differentiated neurons and astrocytes more than unmodified ones. IGF-1 accelerated NSC differentiation but not cell-type differentiation. GO, which may be directly deposited on PLGA surfaces, protects NSCs from ROS-mediated cell death and promotes their differentiation into neurons [130]. PLGA is widely used to manufacture scaffolds for tissue-engineering applications. Patel et al. prepared a PLGA scaffold containing conductive polypyrrole (PPy) layers and microgrooves. In comparison to a nonconductive surface, mNSCs were better aligned and extended on those with microgroove patterns. The PPy layers improved cell interaction with the surface by supporting more and longer filopodia. The microgrooves and the conductive PPy layers improved mNSC neuronal development even without electrical stimulation, and a significant electrical pulse (1.0 V) enhanced it further [131]. Recent research has evaluated the in vitro and in vivo regeneration abilities of PLGA-PEG micelles containing Reelin and embryonic NSCs in a mouse model of a photothrombotic stroke. The Reelin-loaded PLGA-PEG + NSCs group showed a significantly higher NSC proliferation rate, neurite outgrowth, and neuronal differentiation compared to the PLGA-PEG + NSCs and Reelin + NSCs groups but decreased astrocytic gliosis and cavity size. Reelin-loaded PLGA-PEG + NSCs improved tissue regeneration and neurological outcomes [132].

MSCs have been found to stimulate angiogenesis [133], stabilize the blood–brain barrier [134], and modulate the immune system [135]. In several animal models of subacute, acute, or chronic ischemic stroke, MSC transplantation has demonstrated therapeutic effects, with encouraging results even in small clinical studies [136,137]. The research showed that both MSCs and neurons could grow and move in the PLGA scaffold and that the scaffold did not impede MSC proliferation or neural development [138]. Mohammadalizadeh et al. prepared SPION-based magnetic PLGA nanofibers and used them as substrates for MSC differentiation. The magnetic biomaterials could stimulate MSCs to differentiate into neurons, and increasing the content of superparamagnetic ferric oxide from 0 to 10% sped up the procedure [139]. PLGA-PEG nanofibers can create the optimal microenvironment, encourage the development of networks around neurons, and enhance synaptogenesis and inter-neuronal communication [140].

## 5. Applications of PLGA-Based Nanostructures in Imaging Diagnosis of Ischemic Stroke

The ideal combination of nanomaterials and magnetic particles can achieve magnetic targeting in drug administration, as well as controllable and accurate drug delivery. It can also provide the necessary conditions for magnetic resonance imaging (MRI). Zhou et al. developed Fe_3_O_4_-based PLGA NPs for molecular imaging of the thrombus during MRI scanning, allowing for both targeted thrombolysis and the real-time monitoring of thrombolysis efficacy [19]. In a recent study, PLGA NPs were functionalized with superparamagnetic iron oxide NPs (SPION) and Cy7.5. The magnetic PLGA capsules were administered to middle cerebral artery occlusion mice under magnetic guidance. The nanocapsule retention and spatial distribution in the brain could be tracked and modified instantly using MRI and fluorescent molecular imaging (FMI). Furthermore, the experiment examined the safety and practicability of magnetic nanocapsules, providing support for their clinical application [141]. A neutrophil-camouflaged magnetic nanoprobe was developed that successfully bonded to inflamed cerebral microvessels in transient middle cerebral artery occlusion (tMCAO) mice. As a method for imaging neuroinflammation, the neutrophil-camouflaged magnetic nanoprobe has proven exceptionally safe and selective [142]. PLGA NPs are too small to be detected by an optical microscope and have limited tracking ability within the body. This issue can also be resolved by combining fluorescent pigments to detect their signals. A study demonstrated that the biological distribution of fluorescent PLGA NPs could be monitored at a subcellular level with high resolution and precision. In addition, PLGA nanocarriers capable of targeting brain endothelial cells could also be identified [143]. Using confocal imaging, PLGA NPs carrying a fluorophore (TAMRA) were beneficial for monitoring PLGA NPs’ biological distribution in the brain parenchyma and BBB permeability following stroke [20]. SOD-loaded PLGA NPs were fluorescence-labelled and injected into mice via the carotid artery. In addition to protecting against ischemia and reperfusion damage, fluorescently labeled NPs could also locate the CA regions of the hippocampus [144]. As imaging agents, perfluorocarbons (PFCS) are essential for detecting the ischemic penumbra after a stroke and monitoring its metabolic status [145,146]. It was established that PFC-loaded PLGA NPs were appropriate for 19F MRI, ultrasound, and fluorescence multimodal imaging and that they had an excellent clinical translational value [147].

## 6. Shortcomings and Limitations

PLGA-based nanostructures are developed gradually, and their performance and synthesis methods are optimized. However, there are still practical issues, such as cumbersome production processes and unscaled supplies of basic materials, that impede their mass production and widespread use. To enhance every technological aspect, reduce costs, and form high-quality production lines for PLGA-based nanostructures, collaboration in multiple disciplines, such as materials, biology, and medicine, is required. Numerous studies have been conducted on PLGA-based nanostructures, but there are no clear guidelines for research design, experimental analysis, or data statistics [12]. Researchers have demonstrated the phenomenon of PLGA-based nanostructures promoting drug therapeutic efficacy, but their specific mechanisms of action have not been investigated in depth. Extensive clinical research has been conducted on PLGA-based nanostructures for tumors [148,149], oral diseases [150,151,152], and cardiovascular diseases [153,154]. However, clinical research on ischemic stroke is extremely limited, focusing on intracranial arterial stents [155]. This is due to the BBB limiting drug delivery, the complex and critical condition of ischemic stroke patients, and the low acceptance by the general public. PLGA is biodegradable and biocompatible, and the final product is non-toxic. However, aggregation between PLGA NPs or immune reactions in the body may occur, and the modified molecules may be toxic. Therefore, it is difficult to maintain safety in a dynamic human-body environment. In addition, the vast majority of research is conducted using animal models or in vitro models, resulting in a paucity of pertinent information about the human body. Nanomedicine has penetrated clinical practice only gradually in recent years, and the long-term adverse effects of nanomaterials injected into the body must still be monitored. Many preclinical studies have proven the efficacy of PLGA-based nanostructures in the treatment of ischemic stroke; consequently, the transition from the laboratory to the clinic requires persistent efforts.

## 7. Conclusions

Nanomedicine has ushered in an exciting era in the diagnosis and treatment of ischemic stroke, which places an immense burden on society and families. PLGA is a nanomaterial with relative safety and a high plasticity widely used in pharmaceutical products and medical devices. In this paper, we review recent research on PLGA-based nanostructures, highlighting their great potential in drug delivery, disease therapy, cell transplantation, and the diagnostic imaging of ischemic stroke. Particularly, PLGA nanocarriers can carry drugs that are hard to transport into the brain across the blood–brain barrier to stroke lesions for targeted therapy. Nevertheless, the vast majority of current studies are preclinical. The safety and reproducibility of the preparation procedure for numerous novel nanoformulations must be improved. There are also practical issues pertaining to production, regulation, and environmental protection that require interdisciplinary cooperation to resolve. It is anticipated that more PLGA-based nanostructures will be utilized in clinical stroke diagnosis and treatment.

## Figures and Tables

**Table 1:** PLGA-based carriers used in drug therapy for ischemic stroke.

Therapeutic Mechanism	Nanocarrier	Loaded Drug	Synthesis Method	Average Diameter (nm)	Research Type	Year of Publication	Refs.
Thrombolytic therapy	PEG-PLGA NPs	t-PA	Single-emulsion solvent diffusion/evaporation	276.20 ± 27.58	In vitro	2019	[63]
	Peptide conjugated PLGA magnetic NPs	rtPA	Single-emulsion solvent evaporation	321.1 ± 26.9	In vivo and in vitro	2020	[64]
	CREKA polypeptide modified PLGA NPs	rtPA, PFH	Carbodiimide method	178.56 ± 1.25	In vivo and in vitro	2022	[65]
	CS-GRGD modified PLGA NPs	Indocyanine green (ICG) complex of urokinase (ICG@uPA)	Double-emulsion solvent evaporation	68 ± 2	In vivo and in vitro	2022	[66]
Anti-oxidative stress and anti-apoptosis	mPEG-bPLGA NPs	Edaravone	Nanoprecipitation	155 ± 2.5	In vitro	2022	[67]
	PLGA NPs	Scutellarin	Nanoprecipitation	187.89 ± 3.42	In vivo and in vitro	2022	[68]
	PLGA NPs	Tanshinone IIA	Nanoprecipitation	91.34 ± 1.3	In vivo and in vitro	2021	[69]
	PLGA NPs	NL-1	Emulsification and solvent evaporation	123.9 ± 17.1	In vivo and in vitro	2022	[70]
	PLGA NPs	Ex-4	Water–oil–water (w/o/w) emulsion solvent evaporation	68 ± 3.2	In vivo and in vitro	2022	[71]
	PEG-PLGA NPs	Curcumin	Modified emulsion diffusion evaporation	71 ± 9.5	In vivo	2019	[72]
Anti-inflammation	RVG29 peptide-modified polyethylene glycol–polylactic acid–glycolic acid copolymer NPs encapsulated in the membrane of neutrophils	Baicalin	Double emulsification	89~130	In vivo and in vitro	2022	[73]
	α-lipoic acid modified PLGA NPs	Cannabidiol	Classic emulsion/solvent evaporation	110.3 ± 3.6	In vivo and in vitro	2022	[74]
	PLGA NPs	Perampanel	Emulsification/ solvent evaporation	216.7	In vitro and in vivo	2022	[75]
	PLGA NPs	PINK1 siRNA	emulsification/ solvent evaporation	-	In vivo and in vitro	2022	[76]
Inhibition of neuroexcitatory toxicity	PLGA NPs	Estradiol	Emulsion diffusion	98 ± 1.9	In vitro	2014	[77]
	PLGA NPs	TIMP-1	Multiple emulsion and solvent evaporation	-	In vitro	2012	[78]
	PLGA NPs	rhEPO	W/o/w emulsion solvent evaporation	42	In vitro	2014	[79]
	PLGA NPs	Indole-3-methanol	Oil-in-water (o/w) emulsion solvent evaporation	61	In vitro	2015	[80]
Supplementing with neurotrophic factor	PLGA NPs	BDNF	Nanoprecipitation	186.6 ± 19.11	In vivo	2020	[81]
	PLGA NPs-HAMC composite	BDNF	W/o/w double-emulsion solvent evaporation	-	In vivo	2019	[82]

## Data Availability

Not applicable.
